# Valid Inference for Two Sample Summary Statistics Mendelian Randomization

**DOI:** 10.1002/sim.70784

**Published:** 2026-10-18

**Authors:** Kai Wang, Grace Z. Wang

**Affiliations:** ^1^ Department of Biostatistics University of Iowa Iowa City Iowa USA; ^2^ Department of Biostatistics Harvard T.H. Chan School of Public Health Boston Massachusetts USA

**Keywords:** generalized method of moments, inverse‐variance weighting, Mendelian randomization, summary statistics, two‐stage least‐squares regression

## Abstract

Mendelian randomization (MR) studies increasingly rely on summary statistics from genome‐wide association studies (GWASs). However, current summary‐data methods treat marginal GWAS effect estimates as independent across instrumental single‐nucleotide polymorphisms (SNPs), which holds only when the SNPs have no effect. Without imposing this independence assumption, we derive expressions for the two‐stage least squares (TSLS), two‐sample TSLS (TSTSLS), and generalized method‐of‐moments estimators together with their variance estimators using only GWAS summary statistics, assuming the instrumental SNPs are in linkage equilibrium. We show that the widely used inverse‐variance weighted (IVW) estimator is generally not equivalent to either TSLS or TSTSLS, contrary to a common claim in the MR literature. We further demonstrate that the conventional IVW standard error is generally inconsistent and provide a consistent alternative. Extensive simulations confirm the theoretical results and reveal that Wald confidence intervals based on summary statistics can be anti‐conservative. An application to publicly available GWAS summary statistics on body mass index and systolic blood pressure illustrates the practical implications of the proposed methodology.

## 1 Introduction

Mendelian randomization (MR) is an instrumental variable (IV) method that employs single nucleotide polymorphisms (SNPs) as IVs. A widely used approach in MR analysis leverages summary statistics from genome‐wide association studies (GWASs), facilitated by the growing number of available GWASs. It can be applied in either a one‐sample or a two‐sample setting [1]. One key advantage of summary data MR is that it does not require individual‐level data and thus avoids privacy concerns related to individual‐level data.

In a one‐sample setting, the instrumental SNPs, the exposure, and the outcome are measured in the same group of individuals. Conversely, in a two‐sample setting, SNP‐exposure associations and SNP‐outcome associations are estimated from two different samples. Traditionally, these samples are assumed to come from the same population, ensuring homogeneous genetic structures [2, 3]. Methods have been developed to assess such heterogeneity between two GWAS studies using only summary statistics [4].

Two‐stage least squares (TSLS) regression is a powerful tool in handling IV variables [5, 6]. It has some nice asymptotic properties like consistency and asymptotic normality. TSLS is originally developed for one‐sample individual‐level data and has been generalized to two‐sample data [7, 8, 9, 10, 11]. Recently, the two‐sample TSLS (TSTSLS) and other two‐sample IV methods have been applied to two‐sample MR [10, 12]. These methods use moment statistics computed from individual‐level data rather than summary statistics from GWASs.

A popular method for two‐sample summary statistics MR is based on the inverse‐variance weighted (IVW) average of Wald ratios [3, 13]. Using SNPs in linkage equilibrium as instruments, this method adopts techniques developed for meta‐analysis, including point estimation and Cochran's Q test for homogeneity, by treating GWAS summary statistics at each SNP as if they were from “independent studies.” Under the NO Measurement Error (NOME) assumption, the variance of a Wald ratio is estimated using “first‐order weights” [3]. To avoid making the NOME assumption, another weighting scheme named “second‐order weights” is proposed [3]. However, the “second‐order weights” approach is still within the “meta‐analysis” framework.

This meta‐analysis approach, although convenient and straightforward, may not be appropriate for summary statistics MR. The effect estimates at two SNPs are dependent even when those SNPs are in linkage equilibrium. Although the dependence among these effect estimates is acknowledged [14], there are still some studies that develop their theory and conduct simulation studies under the critical assumption that these effect estimates are mutually independent [14, 15, 16]. This practice seems to be continuing [17].

Zhao et al. [12] applied the generalized method of moments (GMM) to the IV analysis using data from two heterogeneous samples. This method encompasses several common two‐sample IV methods, including TSTSLS, as special cases. It relies on moment statistics computed from the individual‐level data and is not designed for summary statistics from GWASs. The variance of their estimator depends on two unknown residual variances [12], Equation ((8) and it is recommended to replace them by the variances of the exposure and the outcome, respectively [12]). Remark 5 Such a solution does not work with GWAS summary statistics as the variances of the exposure and the outcome are not available from a GWAS.

Under the assumption that the instrumental SNPs are in linkage equilibrium, we derive expressions for the TSLS, the TSTSLS, and the GMM estimators as well as their respective asymptotic variances in terms of GWAS summary statistics. Furthermore, we study the property of the IVW method in comparison with the TSTSLS method.

We have several interesting findings. There is a long‐standing claim in the MR literature that the IVW estimate is (asymptotically) equivalent to the TSLS method [18, 19]. However, our results indicate that this claim is misleading. In one‐sample setting, the standard error (SE) of the TSLS estimator even cannot be computed from GWAS summary statistics. In two‐sample setting, the TSLS estimator doesn't apply, and one has to use a two‐sample MR method such as TSTSLS. We show that the current estimate of the SE of an IVW estimator is inconsistent when the effect size is non‐zero, and provide a consistent estimate of the SE of the IVW.

The issue with the IVW method arises from two fallacies: The NOME assumption ignores part of the uncertainties in the summary statistics. The second fallacy comes from not distinguishing the marginal residual variances, there is one for each instrumental SNP, from the residual variance of a linear regression that jointly uses all instrumental SNPs. The second fallacy appears in other methods based on GWAS summary statistics as well, for instance, dIVW [15].

In what follows, we begin with a brief introduction to TSLS method in one‐sample setting. We then address a two‐sample setting, allowing for heterogeneity between the two samples. We present expressions for the TSLS estimator, the TSTSLS estimator, and the optimal GMM method in terms of GWAS summary statistics. In the simulation study, we compare our summary‐data‐based estimates with the IVW estimate and those directly computed from individual‐level data. These estimates are then applied to publicly available GWAS summary statistics on the causal effect of one's body mass index on systolic blood pressure. We conclude with some final remarks.

## 2 Method

We are interested in estimating the causal effect b of an exposure x on an outcome y. The model may be represented as follows:

(1) y=bx+ϵ,

where ϵ represents unobserved factors, some of which may be correlated with x. Without loss of generality, all variables are centered so that E(y)=E(x)=E(ϵ)=0. The variance of ϵ is denoted by $\sigma_{\epsilon }^{2}$. It is well‐known that the ordinary least squares (OLS) estimate of b is biased because Cov(x,ϵ)≠0.

Instrumental variable (IV) method is an effective way to produce a consistent estimate of b. An IV, denoted by z, is exogenous and satisfies the following three assumptions [20, 21].

Assumption 1 (Relevance) It is associated with exposure x (i.e., Cov(z,x)≠0).

Assumption 2 (Exclusion Restriction) It affects outcome y only through its association with exposure x.

Assumption 3 (Exchangeability) It is not associated with any confounders of the exposure‐outcome association (i.e., Cov(z,ϵ)=0).

In MR, an IV is typically a SNP. There are often multiple instrumental SNPs. The number of instrumental SNPs is denoted by p and the p IVs are denoted by $z_{j},j=1,\ldots ,p$.

### 2.1 One‐sample Setting

Let x, y, and $z_{j}$ be n×1 vectors of observed values on x,y and $z_{j},j=1,\ldots ,p$, respectively, where n is the sample size. They are centered so that $1^{\prime }x=1^{\prime }y=1^{\prime }z_{j}=0,j=1,\ldots ,p$.

The TSLS estimator is defined for individual‐level data from a sample. Define $Z=[z_{1},\ldots ,z_{p}]$. Consider the following linear regression,

(2) $$x=Z\gamma +u_{x},$$

where γ is a vector of regression coefficients and the components of the error vector $u_{x}$ are independent and identically distributed according to a normal distribution with mean 0 and variance $\sigma_{x}^{2}$. Equations (1) and (2) constitute the structural equations. A diagram of them is shown in Figure 1.

**FIGURE 1:**
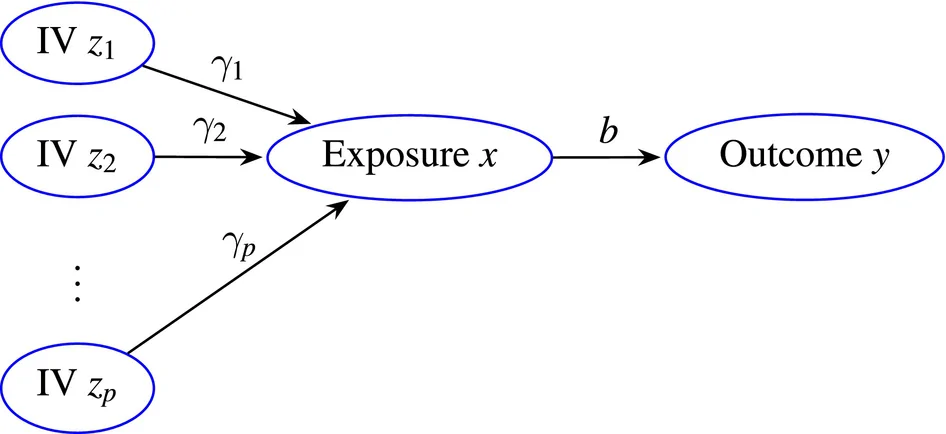
A diagram for the structural models. The instrumental SNPs jointly affect the exposure and the exposure affects the outcome.

From Equations (1) and (2), we have a reduced form equation for y:

(3) $$y=Z\Gamma +u_{y},$$

where Γ=bγ and $u_{y}=bu_{x}+\epsilon$ with ϵ the vector of errors for Equation (1). Equations (2) and (3) constitute the reduced form equations. A diagram for them is shown in Figure 2.

**FIGURE 2:**
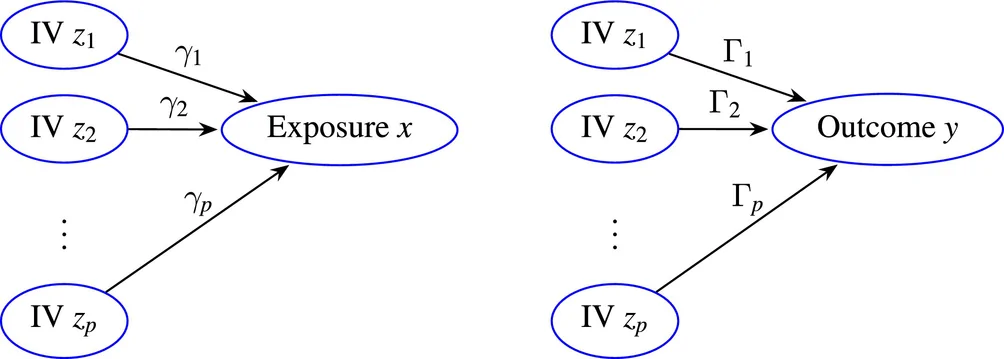
A diagram for the reduced form models where $\Gamma_{j}=b\gamma_{j},j=1,\ldots ,p$. The instrumental SNPs jointly affect the exposure and they also jointly affect the outcome.

Let $\tilde{\gamma }={\left(Z^{\prime }Z\right)}^{-1}Z^{\prime }x$ be the least squares estimator of γ and $\tilde{x}=Z\tilde{\gamma }$ be the predicted vector of x based on Equation (2). It is easy to see that $\tilde{x}$ satisfies $1^{\prime }\tilde{x}=0$, i.e., it is also centered. In TSLS, $\tilde{x}$ is used as an IV and the TSLS estimate of the causal effect b in Equation (1) is given by [6]:

$${\tilde{b}}_{\text{TSLS}}=\frac{{\tilde{x}}^{\prime }y}{{\tilde{x}}^{\prime }\tilde{x}}.$$

${\tilde{b}}_{\text{TSLS}}$ can be written

$${\tilde{b}}_{\text{TSLS}}=\frac{{\tilde{\gamma }}^{\prime }(Z^{\prime }Z)\tilde{\Gamma }}{{\tilde{\gamma }}^{\prime }(Z^{\prime }Z)\tilde{\gamma }},$$

where $\tilde{\Gamma }={\left(Z^{\prime }Z\right)}^{-1}Z^{\prime }y$ is the least squares estimator of Γ.

According to the standard theory on the TSLS estimator [6], ${\tilde{b}}_{\text{TSLS}}$ is consistent and, as n→∞,

$$n^{1/2}({\tilde{b}}_{\text{TSLS}}-b)\overset{d}{\to }N\left(0,\sigma_{\epsilon }^{2}\underset{n\to \infty }{\lim}n{\left({\tilde{x}}^{\prime }\tilde{x}\right)}^{-1}\right).$$

A consistent estimate of $\sigma_{\epsilon }^{2}$ is

(4) $${\tilde{\sigma }}_{\epsilon }^{2}=n^{-1}{\left(y-{\tilde{b}}_{\text{TSLS}}x\right)}^{\prime }(y-{\tilde{b}}_{\text{TSLS}}x).$$

Therefore, a consistent estimate of $\mathrm{Var}\;({\tilde{b}}_{\text{TSLS}})$ is ${\tilde{\sigma }}_{\epsilon }^{2}{\left({\tilde{x}}^{\prime }\tilde{x}\right)}^{-1}$.

Remark 1 ${\tilde{\sigma }}_{\epsilon }^{2}$ is different from $n^{-1}{\left(y-{\tilde{b}}_{\text{TSLS}}\tilde{x}\right)}^{\prime }(y-{\tilde{b}}_{\text{TSLS}}\tilde{x})$, an estimate of the variance of the residuals in a least squares regression where the regressor is $\tilde{x}$ [6]. In other words, replacing x by $\tilde{x}$ in the Equation (4) does not result in a consistent estimator for $\sigma_{\epsilon }^{2}$.

Calculating ${\tilde{\sigma }}_{\epsilon }^{2}$ requires knowing $x^{\prime }y$, a quantity unavailable from GWAS summary statistics. This implies that ${\tilde{\sigma }}_{\epsilon }^{2}$ cannot be calculated from GWAS summary statistics. Furthermore, ${\tilde{b}}_{\text{TSLS}}$ cannot be calculated from summary statistics either because $Z^{\prime }Z$ is unknown. In order to calculate ${\tilde{b}}_{\text{TSLS}}$, we make the following assumption.

Assumption 4 (Orthogonality) There is $Z^{\prime }Z=D$, where $D=\text{diag}[z_{1}^{\prime }z_{1},\cdots \;,z_{p}^{\prime }z_{p}]$.

Under this assumption, $z_{1},\ldots ,z_{p}$ are mutually uncorrelated. This assumption is made because the instrumental SNPs are typically chosen to be in linkage equilibrium. Hence this assumption is expected to hold at least asymptotically. Next we consider how to calculate ${\tilde{b}}_{\text{TSLS}}$ from GWAS summary statistics under Assumption 4.

Let $\hat{\gamma }$ and $\hat{\Gamma }$ be vectors of the estimated marginal regression coefficients for the SNP‐exposure association and the SNP‐outcome association, respectively. There are $\hat{\gamma }=D^{-1}Z^{\prime }x$ and $\hat{\Gamma }=D^{-1}Z^{\prime }y$. These marginal estimates are equal to the joint estimates under Assumption 4: $\hat{\gamma }=\tilde{\gamma },\hat{\Gamma }=\tilde{\Gamma }$. The statistic ${\tilde{b}}_{\text{TSLS}}$ under Assumption 4 is denoted by ${\hat{b}}_{\text{TSLS}}$. Therefore,

$${\hat{b}}_{\text{TSLS}}=\frac{{\hat{\gamma }}^{\prime }D\hat{\Gamma }}{{\hat{\gamma }}^{\prime }D\hat{\gamma }}=\frac{\sum_{j}{\hat{\gamma }}_{j}{\hat{\Gamma }}_{j}z_{j}^{\prime }z_{j}}{\sum_{j}{\hat{\gamma }}_{j}^{2}z_{j}^{\prime }z_{j}}.$$

To calculate ${\hat{b}}_{\text{TSLS}}$, it is sufficient to know the relative size of $z_{j}^{\prime }z_{j},j=1,\ldots ,p$. Wang and Wang [4] proposed a way to calculate their relative sizes from GWAS summary statistics, which is summarized below.

Define $P_{j}=z_{j}{\left(z_{j}^{\prime }z_{j}\right)}^{-1}z_{j}^{\prime }$. We first consider Equation (2) for SNP‐exposure association. Denote the j th component of $\hat{\gamma }$ by ${\hat{\gamma }}_{j}$. By standard least squares theory, the variance of ${\hat{\gamma }}_{j}$ is estimated by

$$\hat{\mathrm{Var}}\;({\hat{\gamma }}_{j})=\frac{x^{\prime }(I-P_{j})x}{\left(n-2\right)}\cdot {\left(z_{j}^{\prime }z_{j}\right)}^{-1},\;\;\;j=1,\ldots ,p.$$

Since $x^{\prime }x=x^{\prime }P_{j}x+x^{\prime }(I-P_{j})x$ and $x^{\prime }P_{j}x={\hat{\gamma }}_{j}^{2}z_{j}^{\prime }z_{j}$, the ratio of the sample variance of $z_{j}$ to that of x, denoted by $v_{x,j}$, is equal to

$$v_{x,j}=\frac{z_{j}^{\prime }z_{j}}{x^{\prime }x}=\frac{1}{{\hat{\gamma }}_{j}^{2}+(n-2)\hat{\mathrm{Var}}\;({\hat{\gamma }}_{j})}.$$

Similarly, the ratio of the sample variance of $z_{j}$ to that of y, denoted by $v_{y,j}$, is

$$v_{y,j}=\frac{z_{j}^{\prime }z_{j}}{y^{\prime }y}=\frac{1}{{\hat{\Gamma }}_{j}^{2}+(n-2)\hat{\mathrm{Var}}\;({\hat{\Gamma }}_{j})},$$

where ${\hat{\Gamma }}_{j}$ is the j th component of $\hat{\Gamma }$. Hence ${\hat{b}}_{\text{TSLS}}$ can be computed either through $\left\{v_{x,j},j=1,\ldots ,p\right\}$ or $\left\{v_{y,j},j=1,\ldots ,p\right\}$:

(5) $${\hat{b}}_{\text{TSLS}}=\frac{\sum_{j}{\hat{\gamma }}_{j}{\hat{\Gamma }}_{j}v_{x,j}}{\sum_{j}{\hat{\gamma }}_{j}^{2}v_{x,j}}=\frac{\sum_{j}{\hat{\gamma }}_{j}{\hat{\Gamma }}_{j}v_{y,j}}{\sum_{j}{\hat{\gamma }}_{j}^{2}v_{y,j}}.$$

Equation (5) gives two equivalent expressions for ${\hat{b}}_{\text{TSLS}}$. Since $v_{x,j},v_{y,j},j=1,\ldots ,p$, depend only on GWAS summary statistics, so does ${\hat{b}}_{\text{TSLS}}$.

Remark 2 In the case that the sample size n varies across instrumental SNPs due to missingness, the sample size n in the expressions for $v_{x,j}$ and $v_{y,j}$ can be replaced by $n_{j}$, the number of genotyped subjects at the j th SNP.

Remark 3 The estimated SNP heritability of the j th SNP for the exposure is equal to ${\hat{\gamma }}_{j}^{2}v_{x,j}={\hat{\gamma }}_{j}^{2}z_{j}^{\prime }z_{j}/x^{\prime }x$. The first expression in Equation (5) can be written

$${\hat{b}}_{\text{TSLS}}=\overset{p}{\underset{j=1}{\sum }}\;\frac{{\hat{\Gamma }}_{j}}{{\hat{\gamma }}_{j}}\cdot \frac{{\hat{\gamma }}_{j}^{2}v_{x,j}}{\sum_{j}{\hat{\gamma }}_{j}^{2}v_{x,j}}.$$

That is, ${\hat{b}}_{\text{TSLS}}$ is a weighted average of the Wald ratios $\left\{{\hat{\Gamma }}_{j}/{\hat{\gamma }}_{j},j=1,\ldots ,p\right\}$ where the weight for the j th SNP is proportional to its SNP heritability for the exposure.

Remark 4 In terms of ${\hat{b}}_{\text{TSLS}}$, a consistent estimate of $\sigma_{\epsilon }^{2}$ is

$${\hat{\sigma }}_{\epsilon }^{2}=n^{-1}{\left(y-{\hat{b}}_{\text{TSLS}}x\right)}^{\prime }(y-{\hat{b}}_{\text{TSLS}}x).$$

Therefore, a consistent estimate of $\mathrm{Var}({\hat{b}}_{\text{TSLS}})$ is $\hat{\mathrm{Var}}\;({\hat{b}}_{\text{TSLS}})={\hat{\sigma }}_{\epsilon }^{2}{\left({\hat{x}}^{\prime }\hat{x}\right)}^{-1}$, where $\hat{x}=Z\hat{\gamma }$ Equation (4). Note that $\hat{\mathrm{Var}}\;({\hat{b}}_{\text{TSLS}})={\hat{\sigma }}_{\epsilon }^{2}{\left({\hat{x}}^{\prime }\hat{x}\right)}^{-1}={\hat{\sigma }}_{\epsilon }^{2}/{\hat{\gamma }}^{\prime }(Z^{\prime }Z)\hat{\gamma }$ cannot be computed from summary statistics: Although the relative magnitudes of $z_{j}^{\prime }z_{j}/$ with respect to $x^{\prime }x$ and $y^{\prime }y$ are known via $v_{x,j}=z_{j}^{\prime }z_{j}/x^{\prime }x$ and $v_{y,j}=z_{j}^{\prime }z_{j}/y^{\prime }y$, there is no such information regarding $x^{\prime }y$.

### 2.2 Two‐sample Setting

The two‐sample IV framework has been extensively investigated in the econometrics literature. Methods developed include the two‐sample IV [7, 8] and the two‐sample TSLS [9, 10, 11]. The two‐sample IV is less efficient than the two‐sample TSLS [10]. These studies, except one [11], assume that the two samples are homogeneous, i.e., the covariance matrix of the IVs in one sample is the same as that in the other. To the best of our knowledge, Zhao et al. [12] is the only publication on MR that addresses heterogeneity between the two samples.

We use subscripts 1 and 2 to distinguish notations for sample 1 and sample 2. In sample 1, we have summary data ${\hat{\Gamma }}_{1}$ and $se({\hat{\Gamma }}_{1})$, and in sample 2, we have summary data ${\hat{\gamma }}_{2}$ and $se({\hat{\gamma }}_{2})$. That is, summary data regarding $\Gamma_{2}$ and $\gamma_{1}$ are unavailable.

To make the causal effect identifiable, the following assumption is made.

Assumption 5 (Structural Invariance) There are Γ and γ so that $\Gamma_{1}=\Gamma_{2}=\Gamma ,\;\;\gamma_{1}=\gamma_{2}=\gamma$.

This is an assumption made by Zhao et al. [12] as well. It implies that these two samples share the same causal effect: $b=\Gamma_{j}/\gamma_{j},j=1,\ldots ,p$, where $\Gamma_{j}$ and $\gamma_{j}$ are the j th elements of Γ and γ, respectively. Therefore, any weighted average of $\left\{{\hat{\Gamma }}_{j}/{\hat{\gamma }}_{j},j=1,\ldots ,p\right\}$ would be a consistent estimator of b as ${\hat{\Gamma }}_{j}$ and ${\hat{\gamma }}_{j}$ are consistent for $\Gamma_{j}$ and $\gamma_{j}$, respectively, for all j=1,…,p. However, an estimate using an *ad hoc* set of weights may not be efficient.

As stated earlier, the TSLS method does not apply to two‐sample MR. A generalization of TSLS to two‐sample setting is to define ${\tilde{x}}_{1}=Z_{1}\tilde{\gamma }$, where $\tilde{\gamma }={\left(Z_{2}^{\prime }Z_{2}\right)}^{-1}Z_{2}^{\prime }x_{2}$ is the least squares estimate of γ in Equation (2) from sample 2. The quantity ${\tilde{x}}_{1}$ is a cross‐sample predictor of $x_{1}$ as $\tilde{\gamma }$ and $Z_{1}$ are from different samples. Using ${\tilde{x}}_{1}$ as an IV in the second stage, the (individual‐level data) TSTSLS estimator is defined as [10, 12]

$${\tilde{b}}_{\text{TSTSLS}}=\frac{{\tilde{x}}_{1}^{\prime }y_{1}}{{\tilde{x}}_{1}^{\prime }{\tilde{x}}_{1}}=\frac{{\tilde{\gamma }}^{\prime }Z_{1}^{\prime }Z_{1}\tilde{\Gamma }}{{\tilde{\gamma }}^{\prime }Z_{1}^{\prime }Z_{1}\tilde{\gamma }},$$

where $\tilde{\Gamma }={\left(Z_{1}^{\prime }Z_{1}\right)}^{-1}Z_{1}^{\prime }y_{1}$. The estimated variance matrices for $\tilde{\Gamma }$ and $\tilde{\gamma }$ are $\hat{\mathrm{Var}}\;(\tilde{\Gamma })={\tilde{\sigma }}_{1,y}^{2}{\left(Z_{1}^{\prime }Z_{1}\right)}^{-1}$ and $\hat{\mathrm{Var}}\;(\tilde{\gamma })={\tilde{\sigma }}_{2,x}^{2}{\left(Z_{2}^{\prime }Z_{2}\right)}^{-1}$, respectively, where ${\tilde{\sigma }}_{1,y}^{2}=n_{1}^{-1}{\left(y_{1}-Z_{1}\tilde{\Gamma }\right)}^{\prime }(y_{1}-Z_{1}\tilde{\Gamma })$ and ${\tilde{\sigma }}_{2,x}^{2}=n_{2}^{-1}{\left(x_{2}-Z_{2}\tilde{\gamma }\right)}^{\prime }(x_{2}-Z_{2}\tilde{\gamma })$ are consistent estimates of the regression residuals for the regression of $y_{1}$ on $Z_{1}$ Equation (3) and the regression of $x_{2}$ on $Z_{2}$ Equation (2), respectively. When Assumption 4 holds for both samples, there are $\tilde{\gamma }=\hat{\gamma }$ and $\tilde{\Gamma }=\hat{\Gamma }$. Furthermore, $\hat{\mathrm{Var}}\;(\tilde{\gamma })$ and $\hat{\mathrm{Var}}\;(\tilde{\Gamma })$ can be computed from GWAS summary statistics as follows.

(6) $$\begin{array}{ll} \hat{\mathrm{Var}}\;(\tilde{\Gamma }) & ={\tilde{\sigma }}_{1,y}^{2}{\left(Z_{1}^{\prime }Z_{1}\right)}^{-1}\\ & =n_{1}^{-1}\left(y_{1}^{\prime }y_{1}-\sum_{j=1}^{p}{\tilde{\Gamma }}_{j}^{2}z_{1,j}^{\prime }z_{1,j}\right)\cdot {\left[\text{diag}(z_{1,1}^{\prime }z_{1,1},\ldots ,z_{1,p}^{\prime }z_{1,p})\right]}^{-1}\\ & =n_{1}^{-1}\left(1-\sum_{j=1}^{p}{\hat{\Gamma }}_{j}^{2}v_{1,y,j}\right)\cdot \text{diag}(v_{1,y,1}^{-1},\ldots ,v_{1,y,p}^{-1}), \end{array}$$

where $v_{1,y,j}=z_{1,j}^{\prime }z_{1,j}/y_{1}^{\prime }y_{1}={\left[{\hat{\Gamma }}_{j}^{2}+(n_{1}-2)\hat{\mathrm{Var}}\;({\hat{\Gamma }}_{j})\right]}^{-1}$. Similarly,

(7) $$\hat{\mathrm{Var}}\;(\tilde{\gamma })=n_{2}^{-1}\left(1-\sum_{j=1}^{p}{\hat{\gamma }}_{j}^{2}v_{2,x,j}\right)\cdot \text{diag}(v_{2,x,1}^{-1},\ldots ,v_{2,x,p}^{-1})$$

where $v_{2,x,j}=z_{2,j}^{\prime }z_{2,j}/x_{2}^{\prime }x_{2}={\left[{\hat{\gamma }}_{j}^{2}+(n_{2}-2)\hat{\mathrm{Var}}\;({\hat{\gamma }}_{j})\right]}^{-1}$. Both $\hat{\mathrm{Var}}\;(\tilde{\gamma })$ and $\hat{\mathrm{Var}}\;(\tilde{\Gamma })$ are diagonal.

It is important to note that, although $\tilde{\gamma }=\hat{\gamma }$ and $\tilde{\Gamma }=\hat{\Gamma }$ under Assumption 4, their variances are different. That is, $\hat{\mathrm{Var}}\;(\tilde{\Gamma })\neq \hat{\mathrm{Var}}\;(\hat{\Gamma })$ and $\hat{\mathrm{Var}}\;(\tilde{\gamma })\neq \hat{\mathrm{Var}}\;(\hat{\gamma })$ asymptotically. Explanations are provided below from two aspects.

First, the diagonal elements are different. In fact, $\hat{\mathrm{Var}}\;({\tilde{\Gamma }}_{j})<\hat{\mathrm{Var}}\;({\hat{\Gamma }}_{j})$ and $\hat{\mathrm{Var}}\;({\tilde{\gamma }}_{j})<\hat{\mathrm{Var}}\;({\hat{\gamma }}_{j})$ asymptotically for j=1,…,p. Let $P_{1,j}=z_{1,j}{\left(z_{1,j}^{\prime }z_{1,j}\right)}^{-1}z_{1,j}^{\prime }$. It is easy to see that $\hat{\mathrm{Var}}\;({\hat{\Gamma }}_{j})={\hat{\sigma }}_{1,y,j}^{2}{\left(z_{1,j}^{\prime }z_{1,j}\right)}^{-1}$, where ${\hat{\sigma }}_{1,y,j}^{2}={\left(n_{1}-2\right)}^{-1}y_{1}^{\prime }(I-P_{1,j})y_{1}$ is the least squares estimate of the residual variance for the marginal regression of $y_{1}$ over SNP j. The subscript j in ${\hat{\sigma }}_{1,y,j}^{2}$ indicates that ${\hat{\sigma }}_{1,y,j}^{2}$ is SNP‐specific. On the other hand, the estimated variance of ${\tilde{\Gamma }}_{j}$ is equal to $\hat{\mathrm{Var}}\;({\tilde{\Gamma }}_{j})={\tilde{\sigma }}_{1,y}^{2}{\left(z_{1,j}^{\prime }z_{1,j}\right)}^{-1}$, where ${\tilde{\sigma }}_{1,y}^{2}=n_{1}^{-1}{\left(y_{1}-Z_{1}\tilde{\Gamma }\right)}^{\prime }(y_{1}-Z_{1}\tilde{\Gamma })$ as before. Rewrite ${\tilde{\sigma }}_{1,y}^{2}$ as

$${\tilde{\sigma }}_{1,y}^{2}=n_{1}^{-1}y_{1}^{\prime }\left(I-\overset{p}{\underset{j=1}{\sum }}P_{1,j}\right)y_{1}.$$

It is apparent that ${\tilde{\sigma }}_{1,y}^{2}<(n_{1}-2)/n_{1}\cdot {\hat{\sigma }}_{1,y,j}^{2}$, which implies $\hat{\mathrm{Var}}\;({\tilde{\Gamma }}_{j})<\hat{\mathrm{Var}}\;({\hat{\Gamma }}_{j})$ asymptotically. Similarly, one can conclude that $\hat{\mathrm{Var}}\;({\tilde{\gamma }}_{j})<\hat{\mathrm{Var}}\;({\hat{\gamma }}_{j})$ asymptotically.

Second, although $\mathrm{Var}(\tilde{\gamma })$ and $\mathrm{Var}(\tilde{\Gamma })$ are diagonal, neither $\mathrm{Var}(\hat{\gamma })$ nor $\mathrm{Var}(\hat{\Gamma })$ is. The correlation between ${\hat{\gamma }}_{j}$ and ${\hat{\gamma }}_{k}$ is [14]

(8) $$Cor^{2}({\hat{\gamma }}_{j},{\hat{\gamma }}_{k})=\frac{r_{j}^{2}}{1-r_{j}^{2}}\cdot \frac{r_{k}^{2}}{1-r_{k}^{2}}\neq 0,$$

where $r_{j}^{2}=\gamma_{j}^{2}\mathrm{Var}(z_{2,j})/\mathrm{Var}(x_{2})$ is the proportion of the variance of $x_{2}$ explained by $z_{2,j}$, the j th IV in sample 2. Zhao et al. [14] erroneously include an additional factor of 4 in this expression. The quantity $r_{j}^{2}$ corresponds to the SNP heritability (Remark 3). Similarly, $Cor^{2}({\hat{\Gamma }}_{j},{\hat{\Gamma }}_{k})\neq 0$. Consequently, $Cor({\hat{\gamma }}_{j},{\hat{\gamma }}_{k})=0$ and $Cor({\hat{\Gamma }}_{j},{\hat{\Gamma }}_{k})=0$ only when $\gamma_{j}=\Gamma_{j}=0,j=1,\ldots ,p$. These correlations are ignored in the literature, sometimes deliberately to simplify the analysis [13, 14, 15].

Zhao et al. [12] proposed a class of two‐sample IV estimators via the GMM. Let W be a prespecified weighting matrix. A GMM estimator, denoted by ${\tilde{b}}_{W}$, is given by

$${\tilde{b}}_{W}=\underset{b}{\text{argmin}}\;{\left(\tilde{\Gamma }-b\tilde{\gamma }\right)}^{\prime }W(\tilde{\Gamma }-b\tilde{\gamma })=\frac{{\tilde{\gamma }}^{\prime }W\tilde{\Gamma }}{{\tilde{\gamma }}^{\prime }W\tilde{\gamma }}.$$

The estimator ${\tilde{b}}_{W}$ is defined for individual‐level data. Unlike conventional GMM settings, the effective sample size is one, in the sense that only a single realization of the vectors $\hat{\Gamma }$ and $\hat{\gamma }$ is observed. The TSTSLS estimator is obtained by setting $W=Z_{1}^{\prime }Z_{1}$ [10, 12]. Because $Z_{1}^{\prime }Z_{1}$ is not available from the GWAS summary statistics, it is recommended that it is estimated from an external reference sample [12]. However, this requirement can be avoided when Assumption 4 holds. Specifically, if Assumption 4 is satisfied in both samples 1 and 2, then the TSTSLS estimator ${\tilde{b}}_{\text{TSTSLS}}$ can be expressed entirely in terms of GWAS summary statistics through $v_{1,y,j},\;j=1,\ldots ,p$. Denote the resulting estimator by ${\hat{b}}_{\text{TSTSLS}}$, we obtain

$${\hat{b}}_{\text{TSTSLS}}=\frac{{\hat{\gamma }}^{\prime }Z_{1}^{\prime }Z_{1}\hat{\Gamma }}{{\hat{\gamma }}^{\prime }Z_{1}^{\prime }Z_{1}\hat{\gamma }}=\frac{\sum_{j}{\hat{\gamma }}_{j}{\hat{\Gamma }}_{j}v_{1,y,j}}{\sum_{j}{\hat{\gamma }}_{j}^{2}v_{1,y,j}}.$$

This expression is analogous to the second expression of ${\hat{b}}_{\text{TSLS}}$ shown in Equation (5).

The variance of ${\hat{b}}_{\text{TSTSLS}}$ can be calculated from GWAS summary statistics under the following assumption.

Assumption 6 (Sample Independence) The outcome GWAS sample (sample 1) is independent of the exposure GWAS sample (sample 2).

There has been some confusion in the literature regarding the calculation of ${\hat{b}}_{\text{TSTSLS}}$ [10, 11]. For clarity, we provide additional details below.

Let $\Omega =\mathrm{Var}(\tilde{\Gamma }-b\tilde{\gamma })$, where b denotes the true causal effect. Assumption 6 ensures that Ω can be estimated from GWAS summary statistics alone. A notable counterexample occurs in the one‐sample setting, where the exposure and outcome GWASs are conducted on the same individuals. In that case, Ω depends on the cross‐sample moment $x^{\prime }y$. Under Assumption 6, however, these cross‐sample terms vanish and $\Omega =\mathrm{Var}(\tilde{\Gamma })+b^{2}\mathrm{Var}(\tilde{\gamma })$. Replacing the unknown parameter b with a consistent estimator, such as ${\tilde{b}}_{\text{TSTSLS}}$, yields the consistent estimator

$$\hat{\Omega }=\hat{\mathrm{Var}}\;(\tilde{\Gamma })+{\tilde{b}}_{\text{TSTSLS}}^{2}\hat{\mathrm{Var}}\;(\tilde{\gamma }).$$

Furthermore,

$${\tilde{b}}_{\text{TSTSLS}}-b=\frac{{\tilde{\gamma }}^{\prime }(Z_{1}^{\prime }Z_{1})(\tilde{\Gamma }-b\tilde{\gamma })}{{\tilde{\gamma }}^{\prime }(Z_{1}^{\prime }Z_{1})\tilde{\gamma }}.$$

Under the additional assumption that there exists a α>0 such that $\lim_{n_{1},n_{2}\to \infty }n_{1}/n_{2}=\alpha$, we have

$$n_{1}\Omega \to \underset{n_{1}\to \infty }{\lim}n_{1}\mathrm{Var}(\tilde{\Gamma })+\alpha b^{2}\underset{n_{2}\to \infty }{\lim}n_{2}\mathrm{Var}(\tilde{\gamma }),\;\;\;\text{as}\;n_{1},n_{2}\to \infty .$$

Since

$$n_{1}^{1/2}(\tilde{\Gamma }-b\tilde{\gamma })\overset{d}{\to }N(0,\underset{n_{1}\to \infty }{\lim}n_{1}\Omega ),$$

the asymptotic variance of ${\tilde{b}}_{\text{TSTSLS}}$ follows directly from the delta method. An estimate of the variance of ${\hat{b}}_{\text{TSTSLS}}$ is given by

$$\hat{\mathrm{Var}}\;({\tilde{b}}_{\text{TSTSLS}})=\hat{\mathrm{Var}}\;({\tilde{b}}_{\text{TSTSLS}}-b)=\frac{{\tilde{\gamma }}^{\prime }(Z_{1}^{\prime }Z_{1})\hat{\Omega }(Z_{1}^{\prime }Z_{1})\tilde{\gamma }}{{\left[{\tilde{\gamma }}^{\prime }(Z_{1}^{\prime }Z_{1})\tilde{\gamma }\right]}^{2}}.$$

Under Assumption 4, ${\tilde{b}}_{\text{TSTSLS}}$ reduces to ${\hat{b}}_{\text{TSTSLS}}$ and $\hat{\mathrm{Var}}\;({\tilde{b}}_{\text{TSTSLS}})$ simplifies to $\hat{\mathrm{Var}}\;({\hat{b}}_{\text{TSTSLS}})$, where

(9) $$\hat{\mathrm{Var}}\;({\hat{b}}_{\text{TSTSLS}})=\frac{\sum_{j}{\hat{\gamma }}_{j}^{2}v_{1,y,j}^{2}\left[\hat{\mathrm{Var}}\;({\tilde{\Gamma }}_{j})+{\hat{b}}_{\text{TSTSLS}}^{2}\hat{\mathrm{Var}}\;({\tilde{\gamma }}_{j})\right]}{{\left(\sum_{j}{\hat{\gamma }}_{j}^{2}v_{1,y,j}\right)}^{2}}.$$

The estimator ${\hat{b}}_{\text{TSTSLS}}$ is generally inefficient because the weighting matrix $W=Z_{1}^{\prime }Z_{1}$ is not optimal. The GMM efficiency result implies that the choice of W minimizing the asymptotic variance of ${\tilde{b}}_{W}$ is proportional to $\Omega^{-1}$ [12]. The resulting optimal two‐sample IV estimator, denoted by ${\hat{b}}_{\text{TSIV}}$, is

$${\hat{b}}_{\text{TSIV}}=\frac{{\tilde{\gamma }}^{\prime }\Omega^{-1}\tilde{\Gamma }}{{\tilde{\gamma }}^{\prime }\Omega^{-1}\tilde{\gamma }}=\frac{{\hat{\gamma }}^{\prime }\Omega^{-1}\hat{\Gamma }}{{\hat{\gamma }}^{\prime }\Omega^{-1}\hat{\gamma }}.$$

Its variance is

$$\mathrm{Var}({\hat{b}}_{\text{TSIV}})=\frac{1}{{\tilde{\gamma }}^{\prime }\Omega^{-1}\tilde{\gamma }},$$

and is estimated by

$$\hat{\mathrm{Var}}\;({\hat{b}}_{\text{TSIV}})=\frac{1}{{\hat{\gamma }}^{\prime }{\hat{\Omega }}^{-1}\hat{\gamma }}.$$

In practice, $\hat{\Omega }$ may be obtained by replacing b in Ω with the consistent estimator. Consequently, ${\hat{b}}_{\text{TSTSLS}}$, ${\hat{b}}_{\text{TSIV}}$, and $\hat{\Omega }$ can all be expressed in terms of GWAS summary statistics. The same is true for $\hat{\mathrm{Var}}\;({\hat{b}}_{\text{TSTSLS}})$ and $\hat{\mathrm{Var}}\;({\hat{b}}_{\text{TSIV}})$.

Remark 5 Zhao et al. [12] used $\hat{\mathrm{Var}}\;(\tilde{\Gamma })$ rather than $\hat{\Omega }$ in their Equation (7). This choice appears to reflect the confusion surrounding the variance calculation for ${\hat{b}}_{\text{TSTSLS}}$. Using $\hat{\mathrm{Var}}\;(\tilde{\Gamma })$ ignores the estimation uncertainty in $\tilde{\gamma }$ and is equivalent to imposing the NOME assumption adopted by the IVW method. Despite this issue, the variance formula for the optimal TSIV given in Equation (9) of Zhao et al. [12] remains correct.

### 2.3 IVW Estimator

The inverse‐variance weighted (IVW) method is arguably the most widely used approach for MR based on summary data. The IVW estimator is defined as [13, 22, 23]:

$${\hat{b}}_{\text{IVW}}=\underset{j}{\sum }w_{j}\frac{{\hat{\Gamma }}_{j}}{{\hat{\gamma }}_{j}},\;\;\;\;w_{j}=\frac{{\hat{\gamma }}_{j}^{2}/\hat{\mathrm{Var}}\;({\hat{\Gamma }}_{j})}{\sum_{j}{\hat{\gamma }}_{j}^{2}/\hat{\mathrm{Var}}\;({\hat{\Gamma }}_{j})}.$$

As before, ${\hat{\Gamma }}_{j}$ denotes the marginal regression coefficient for the association between SNP j and the outcome, whereas ${\hat{\gamma }}_{j}$ denotes the marginal regression coefficient for the association between SNP j and the exposure. In addition,

$$\hat{\mathrm{Var}}\;({\hat{\Gamma }}_{j})={\left(n_{1}-2\right)}^{-1}y_{1}^{\prime }(I-P_{j})y_{1}\cdot {\left(z_{j}^{\prime }z_{j}\right)}^{-1}.$$

Under the NOME assumption the estimated variance of ${\hat{b}}_{\text{IVW}}$ is given by [13, 19]

(10) $${\hat{\mathrm{Var}}}_{\text{naïve}}({\hat{b}}_{\text{IVW}})=\sum_{j}\frac{w_{j}^{2}}{{\hat{\gamma }}_{j}^{2}}\mathrm{Var}({\hat{\Gamma }}_{j})={\left(\sum_{j}\frac{{\hat{\gamma }}_{j}^{2}}{\hat{\mathrm{Var}}\;({\hat{\Gamma }}_{j})}\right)}^{-1}.$$

The quantity ${\hat{\mathrm{Var}}}_{\text{naïve}}({\hat{b}}_{\text{IVW}})$ is referred to as the naïve variance estimator of IVW.

It is often asserted that ${\hat{b}}_{\text{IVW}}$ and ${\hat{b}}_{\text{TSLS}}$ are asymptotically equivalent [18, 19]. However, it is unclear whether this assertion refers to the one‐sample setting, the two‐sample setting, or both. In fact, the claim is misleading in either context.

In one‐sample setting, the variance of ${\hat{b}}_{\text{TSLS}}$ cannot be computed from GWAS summary statistics alone (Remark 4). By contrast, the variance estimator for ${\hat{b}}_{\text{IVW}}$ in Equation (10), depends only on GWAS summary statistics. Therefore, the two variance estimators cannot coincide, implying that ${\hat{b}}_{\text{TSLS}}$ and ${\hat{b}}_{\text{IVW}}$ are not equivalent, even asymptotically. In the two‐sample setting, ${\hat{b}}_{\text{TSLS}}$ is not defined, so the question of its equivalence to ${\hat{b}}_{\text{IVW}}$ does not arise.

Because ${\hat{b}}_{\text{TSTSLS}}$ extends ${\hat{b}}_{\text{TSLS}}$ to the two‐sample setting, one might suspect that the claimed equivalence instead concerns ${\hat{b}}_{\text{IVW}}$ and ${\hat{b}}_{\text{TSTSLS}}$. However, no such equivalence holds. First,

$${\hat{b}}_{\text{TSTSLS}}=\frac{\sum_{j}{\hat{\gamma }}_{j}{\hat{\Gamma }}_{j}{\left[{\hat{\Gamma }}_{j}^{2}+(n_{1}-2)\hat{\mathrm{Var}}\;({\hat{\Gamma }}_{j})\right]}^{-1}}{\sum_{j}{\hat{\gamma }}_{j}^{2}{\left[{\hat{\Gamma }}_{j}^{2}+(n_{1}-2)\hat{\mathrm{Var}}\;({\hat{\Gamma }}_{j})\right]}^{-1}},$$

under Assumption 6. Thus, although ${\hat{b}}_{\text{TSTSLS}}$, like ${\hat{b}}_{\text{IVW}}$, can be expressed as a weighted average of Wald ratios ${\hat{\Gamma }}_{j}/{\hat{\gamma }}_{j},j=1,\ldots ,p$, the two estimators employ different weighting schemes and are therefore generally distinct. Indeed, ${\hat{b}}_{\text{TSTSLS}}={\hat{b}}_{\text{IVW}}$ only under highly restrictive conditions, such as ${\hat{\Gamma }}_{j}=0,\;j=1,\ldots ,p$.

The variance estimators are also different. The variance formula commonly reported for ${\hat{b}}_{\text{IVW}}$ under the NOME condition, given in Equation (10), is generally inconsistent. To demonstrate this, we derive a consistent variance estimator below.

Analogous to the derivation of $\mathrm{Var}({\hat{b}}_{\text{TSTSLS}})$ in the previous subsection, the variance of ${\hat{b}}_{\text{IVW}}$ is given by

(11) $$\begin{array}{ll} \mathrm{Var}({\hat{b}}_{\text{IVW}}) & =\mathrm{Var}({\hat{b}}_{\text{IVW}}-b)\\ & =\underset{j}{\sum }\frac{w_{j}^{2}}{{\hat{\gamma }}_{j}^{2}}\mathrm{Var}({\tilde{\Gamma }}_{j}-b{\tilde{\gamma }}_{j})+o_{p}(n_{1}^{-1})\\ & =\underset{j}{\sum }\frac{w_{j}^{2}}{{\hat{\gamma }}_{j}^{2}}\left[\mathrm{Var}({\tilde{\Gamma }}_{j})+b^{2}\mathrm{Var}({\tilde{\gamma }}_{j})\right]+o_{p}(n_{1}^{-1}), \end{array}$$

where the second equality follows from the delta method and the identities ${\tilde{\Gamma }}_{j}={\hat{\Gamma }}_{j}$ and ${\tilde{\gamma }}_{j}={\hat{\gamma }}_{j}$, which hold under Assumption 4.

A natural question is why $\mathrm{Var}({\tilde{\Gamma }}_{j}-b{\tilde{\gamma }}_{j})$ appears in Equation (11) rather than $\mathrm{Var}({\hat{\Gamma }}_{j}-b{\hat{\gamma }}_{j})$. The reason is that the variance calculation must be conditioned on the complete set of instruments, namely $Z_{1}$ and $Z_{2}$. By the law of total variance,

$$\begin{array}{ll} \mathrm{Var}({\tilde{\Gamma }}_{j}-b{\tilde{\gamma }}_{j}) & =E\left[\mathrm{Var}({\tilde{\Gamma }}_{j}-b{\tilde{\gamma }}_{j}|Z_{1},Z_{2})\right]\\ & \;+\mathrm{Var}\left[E({\tilde{\Gamma }}_{j}-b{\tilde{\gamma }}_{j}|Z_{1},Z_{2})\right]. \end{array}$$

Since

$$E({\tilde{\Gamma }}_{j}-b{\tilde{\gamma }}_{j}|Z_{1},Z_{2})=0,$$

the second term vanishes, yielding

$$\begin{array}{ll} \mathrm{Var}({\tilde{\Gamma }}_{j}-b{\tilde{\gamma }}_{j}) & =E\left[\mathrm{Var}({\tilde{\Gamma }}_{j}|Z_{1},Z_{2})\right]+b^{2}E\left[\mathrm{Var}({\tilde{\gamma }}_{j}|Z_{1},Z_{2})\right]\\ & =E\left[\mathrm{Var}({\tilde{\Gamma }}_{j}|Z_{1})\right]+b^{2}E\left[\mathrm{Var}({\tilde{\gamma }}_{j}|Z_{2})\right]. \end{array}$$

Consequently, a consistent estimator of $\mathrm{Var}({\tilde{\Gamma }}_{j}-b{\tilde{\gamma }}_{j})$ is

$$\hat{\mathrm{Var}}\;({\tilde{\Gamma }}_{j}-b{\tilde{\gamma }}_{j})=\hat{\mathrm{Var}}\;({\tilde{\Gamma }}_{j}|Z_{1})+b^{2}\hat{\mathrm{Var}}\;({\tilde{\gamma }}_{j}|Z_{2}).$$

Since

$$\hat{\mathrm{Var}}\;({\tilde{\Gamma }}_{j}|Z_{1})=\hat{\mathrm{Var}}\;({\tilde{\Gamma }}_{j}),\;\;\;\hat{\mathrm{Var}}\;({\tilde{\gamma }}_{j}|Z_{2})=\hat{\mathrm{Var}}\;({\tilde{\gamma }}_{j}),$$

by definition, Equation (11) leads to the consistent variance estimator

$$\hat{\mathrm{Var}}\;({\hat{b}}_{\text{IVW}})=\underset{j}{\sum }\frac{w_{j}^{2}}{{\hat{\gamma }}_{j}^{2}}\left[\hat{\mathrm{Var}}\;({\tilde{\Gamma }}_{j})+{\hat{b}}^{2}\hat{\mathrm{Var}}\;({\tilde{\gamma }}_{j})\right],$$

where $\hat{b}$ denotes any consistent estimator of b such as ${\hat{b}}_{\text{TSTSLS}}$ or ${\hat{b}}_{\text{IVW}}$.

The naïve variance estimator in Equation (10) is consistent only when b=0. In that case, $\hat{\mathrm{Var}}\;({\tilde{\Gamma }}_{j})$ and $\hat{\mathrm{Var}}\;({\hat{\Gamma }}_{j})$ converge to the same value for all j=1,…,p. Therefore, the commonly stated asymptotic equivalence between IVW and TSLS lacks theoretical support in general.

How do the naïve and consistent variance estimators compare? Comparing Equations (10) and (11), we see that the key distinction lies between $\mathrm{Var}({\hat{\Gamma }}_{j})$ and $\mathrm{Var}({\tilde{\Gamma }}_{j})+b^{2}\mathrm{Var}({\tilde{\gamma }}_{j})$. When b=0,

$$Var_{\text{naïve}}({\hat{b}}_{\text{IVW}})>\mathrm{Var}({\hat{b}}_{\text{IVW}}),$$

because $\mathrm{Var}({\hat{\Gamma }}_{j})>\mathrm{Var}({\tilde{\Gamma }}_{j})$. By continuity, the inequality may continue to hold when |b| is sufficiently small. In such cases, the naïve variance estimator overestimates the true variance and therefore leads to conservative inference.

However, when |b| becomes large, it is possible that

$$\mathrm{Var}({\hat{\Gamma }}_{j})<\mathrm{Var}({\tilde{\Gamma }}_{j})+b^{2}\mathrm{Var}({\tilde{\gamma }}_{j}),\;\;\;j=1,\ldots ,p,$$

even though $\mathrm{Var}({\hat{\Gamma }}_{j})>\mathrm{Var}({\tilde{\Gamma }}_{j})$, in which case

$$Var_{\text{naïve}}({\hat{b}}_{\text{IVW}})<\mathrm{Var}({\hat{b}}_{\text{IVW}}).$$

The naïve variance estimator then understates the true uncertainty, leading to anti‐conservative inference and an inflated false‐positive rate. Numeric evidence of this phenomenon is provided in the Simulation Study section.

The “second‐order weights” [3] uses $\mathrm{Var}({\hat{\Gamma }}_{j})+b^{2}\mathrm{Var}({\hat{\gamma }}_{j})$ rather than $\mathrm{Var}({\tilde{\Gamma }}_{j})+b^{2}\mathrm{Var}({\tilde{\gamma }}_{j})$ in Eq. (11). Such a weighting scheme apparently results in a larger variance estimator.

The IVW estimator is not unique in failing to condition on the full set of instruments when estimating variance. Several summary‐data MR methods, including IVW, dIVW [15], and the “exact” weight IVW [3], rely on analogous variance calculations and therefore suffer from the same issue. To the best of our knowledge, this problem has not been explicitly recognized in the Mendelian randomization literature.

### 2.4 Covariates

So far, we have assumed that neither the exposure GWAS nor the outcome GWAS includes covariates. When covariates are included, however, ${\hat{b}}_{\text{TSTSLS}}$, ${\hat{b}}_{\text{TSIV}}$, and their corresponding standard errors can still be expressed in terms of GWAS summary statistics, provided that the assumptions are modified appropriately. We illustrate this extension using ${\hat{b}}_{\text{TSTSLS}}$.

Let $C_{1}$ be the $n_{1}\times k_{1}$ matrix of $k_{1}$ covariates in the outcome GWAS and $C_{2}$ be the $n_{2}\times k_{2}$ matrix of $k_{2}$ covariates in the exposure GWAS. Accordingly, the $k_{1}$ covariates enter the outcome model in Equation (1). Define $M_{1}=I-C_{1}{\left(C_{1}^{\prime }C_{1}\right)}^{-1}C_{1}^{\prime }$. After partialling out the effects of these covariates, Equation (3) for sample 1 becomes

$$M_{1}y_{1}=M_{1}Z_{1}\Gamma +u_{y_{1}},$$

as $M_{1}u_{y_{1}}=u_{y_{1}}$ under uncorrelatedness between $C_{1}$ and $u_{y_{1}}$. Similarly, Equation (2) for sample 2 becomes

$$M_{2}x_{2}=M_{2}Z_{2}\gamma +u_{x_{2}},$$

where $M_{2}=I-C_{2}{\left(C_{2}^{\prime }C_{2}\right)}^{-1}C_{2}^{\prime }$. It follows that

$$\tilde{\gamma }={\left(Z_{2}^{\prime }M_{2}Z_{2}\right)}^{-1}Z_{2}^{\prime }M_{2}x_{2},\;\;\;\tilde{\Gamma }={\left(Z_{1}^{\prime }M_{1}Z_{1}\right)}^{-1}Z_{1}^{\prime }M_{1}y_{1}.$$

The cross‐sample predictor for $M_{1}x_{1}$ is $M_{1}Z_{1}\tilde{\gamma }$. The TSTSLS estimate of the causal effect b becomes

$${\tilde{b}}_{\text{TSTSLS}}=\frac{{\tilde{\gamma }}^{\prime }Z_{1}^{\prime }M_{1}y_{1}}{{\tilde{\gamma }}^{\prime }Z_{1}^{\prime }M_{1}Z_{1}\tilde{\gamma }}=\frac{{\tilde{\gamma }}^{\prime }Z_{1}^{\prime }M_{1}Z_{1}\tilde{\Gamma }}{{\tilde{\gamma }}^{\prime }Z_{1}^{\prime }M_{1}Z_{1}\tilde{\gamma }}.$$

The GWAS estimate of Γ controlling for $C_{1}$ is

$$\hat{\Gamma }=D_{1}^{-1}Z_{1}^{\prime }M_{1}x_{1},\;\;\;D_{1}=diag(Z_{1}M_{1}Z_{1}).$$

Likewise, the GWAS estimate of γ adjusted for $C_{2}$ is

$$\hat{\gamma }=D_{2}^{-1}Z_{2}^{\prime }M_{2}x_{2},\;\;\;D_{2}=diag(Z_{2}M_{2}Z_{2}).$$

If the off‐diagonal elements of $Z_{1}^{\prime }M_{1}Z_{1}$ and $Z_{2}^{\prime }M_{2}Z_{2}$ are assumed to be zero, which is a covariate‐adjusted version of Assumption 6, then $\tilde{\Gamma }=\hat{\Gamma }$ and $\tilde{\gamma }=\hat{\gamma }$. Consequently, ${\tilde{b}}_{\text{TSTSLS}}$ reduces to

$${\hat{b}}_{\text{TSTSLS}}=\frac{{\tilde{\gamma }}^{\prime }D_{1}\tilde{\Gamma }}{{\tilde{\gamma }}^{\prime }D_{1}\tilde{\gamma }}=\frac{\sum_{j}{\hat{\gamma }}_{j}{\hat{\Gamma }}_{j}v_{1,y,j}}{\sum_{j}{\hat{\gamma }}_{j}^{2}v_{1,y,j}},$$

where

$$v_{1,y,j}=\frac{z_{1,j}^{\prime }M_{1}z_{1,j}}{y_{1}^{\prime }M_{1}y_{1}}={\left[{\hat{\Gamma }}_{j}^{2}+(n_{1}-2-k_{1})\mathrm{Var}({\hat{\Gamma }}_{j})\right]}^{-1}$$

is the j th element of $D_{1}{\left(y_{1}^{\prime }M_{1}y_{1}\right)}^{-1}$. The quantity $\left(n_{1}-2-k_{1}\right)$ appears because, in addition to the intercept and the j th SNP, the regression includes $k_{1}$ covariates. With these modifications, the expression for $\hat{\mathrm{Var}}\;(\tilde{\Gamma })$ remains unchanged and the same is true for $\hat{\mathrm{Var}}\;(\tilde{\gamma })$. That is, the variance formulas given in Equations (6) and (7) continue to apply. In particular, the variance estimator in Equation (9) remains consistent.

## 3 Simulation Study

A simulation study was conducted to evaluate the theoretical findings developed in this paper. Some previous studies generated ${\hat{\gamma }}_{j}$ and ${\hat{\Gamma }}_{j}$ independently from normal distributions [14, 15, 16, 17]. However, data generated in this manner do not represent genuine GWAS summary statistics. As emphasized throughout this paper, summary statistics obtained from a GWAS are generally correlated rather than independent. Therefore, valid GWAS summary statistics should be generated from an underlying data‐generating model rather than simulated independently.

The generating model we use is similar to the model considered elsewhere [24] and is given by

(12) $$\left\{\begin{array}{lll} \gamma_{j} & = & j/10,j=1,\ldots ,10,\\ z_{j} & = & \text{Binomial}(2,0.3)\;\text{independently across}\;j=1,\ldots ,10,\\ u & = & \text{Normal}(0,2),\\ \epsilon & = & \text{Normal}(0,2),\\ x & = & \overset{10}{\underset{j=1}{\sum }}\gamma_{j}z_{j}+u+\epsilon ,\\ y & = & bx+\epsilon , \end{array}\right..$$

where u and ϵ are independent. GWAS summary statistics are calculated from the generated data.

Under this model, Var(x)=5.617. Collectively, the ten SNPs $z_{1},\ldots ,z_{10}$ explain 28.79% of the variance in x, while the proportion explained by an individual SNP ranges from 0.07% to 7.48%. Furthermore,

$$\mathrm{Var}(y)=b^{2}\mathrm{Var}(x)+2b\cdot Cov(x,\epsilon )+\mathrm{Var}(\epsilon )=5.617b^{2}+4b+2.$$

For b=0.4, Var(y)=4.49872. In this case, the ten SNPs jointly explain 5.75% of the variance in y, while the proportion explained by an individual SNP ranges from 0.01% to 1.49%.

One reviewer expressed interest in the magnitude of $Cor({\hat{\gamma }}_{j},{\hat{\gamma }}_{k})$ and $Cor({\hat{\Gamma }}_{j},{\hat{\Gamma }}_{k})$. To investigate this, we estimated these correlations from 1000 datasets generated under model (12), each with a sample size of 10,000. We obtain $\hat{Cor}({\hat{\gamma }}_{1},{\hat{\gamma }}_{5})=0.015$ and $\hat{Cor}({\hat{\Gamma }}_{1},{\hat{\Gamma }}_{5})=0.033$ for b=0.4. These values are of the same order of magnitude as the theoretical correlations implied by Equation (8): $Cor({\hat{\gamma }}_{1},{\hat{\gamma }}_{5})=0.004$ and $Cor({\hat{\Gamma }}_{1},{\hat{\Gamma }}_{5})=0.004$, considering the observed substantial variability in the simulated correlations. For example, $\hat{Cor}({\hat{\gamma }}_{1},{\hat{\gamma }}_{6})=0.003$, which is smaller than $\hat{Cor}({\hat{\gamma }}_{1},{\hat{\gamma }}_{6})$, even though the corresponding theoretical correlation is larger according to Equation (8).

### 3.1 One‐sample Setting

In this setting, 1000 datasets are generated from model (12), each with a sample size of 10,000. For each dataset, ${\tilde{b}}_{\text{TSLS}}$ and ${\hat{b}}_{\text{IVW}}$ are computed, and their standard errors are recorded. These reported standard errors are averages across the 1000 simulation replicates. The estimator ${\tilde{b}}_{\text{TSLS}}$ and its standard error are obtained from individual‐level data using the function ivreg in the R package ivreg. The estimator ${\hat{b}}_{\text{IVW}}$ is computed from GWAS summary statistics, and its standard error is computed using Equation (10). The empirical standard deviations of ${\tilde{b}}_{\text{TSLS}}$ and ${\tilde{b}}_{\text{TSLS}}$ across the 1000 datasets are also calculated. The simulation results are presented in Table 1.

**TABLE 1:** Simulation results under one‐sample setting with sample size n=10,000 over 1,000 replicates.

*b*	Estimator	Estimate	SE^a^	SD^b^
0.0	${\tilde{b}}_{\text{TSLS}}$	0.0004	0.0111	0.0113
	${\hat{b}}_{\text{IVW}}$	0.0004	0.0111	0.0113
0.2	${\tilde{b}}_{\text{TSLS}}$	0.2004	0.0111	0.0113
	${\hat{b}}_{\text{IVW}}$	0.2005	0.0136	0.0113
0.4	${\tilde{b}}_{\text{TSLS}}$	0.4004	0.0111	0.0113
	${\hat{b}}_{\text{IVW}}$	0.4005	0.0166	0.0113
0.8	${\tilde{b}}_{\text{TSLS}}$	0.8004	0.0111	0.0113
	${\hat{b}}_{\text{IVW}}$	0.8005	0.0230	0.0113

^a^ The SE of ${\hat{b}}_{\text{IVW}}$ is the naïve SE computed using Equation (10).

^b^ The standard deviation across 1000 replicates.

As shown in Table 1, both ${\tilde{b}}_{\text{TSLS}}$ and ${\hat{b}}_{\text{IVW}}$ are very close to the true value of b across all settings, consistent with the fact that both estimators are consistent. The key difference lies in their estimated standard errors. For every value of b, the average standard error of ${\hat{b}}_{\text{IVW}}$ tends to exceed that of ${\tilde{b}}_{\text{TSLS}}$, with the discrepancy reaching nearly a factor of two when b=0.8. This finding is consistent with previous reports that confidence intervals based on ${\hat{b}}_{\text{IVW}}$ may exhibit inaccurate coverage probabilities [25, 26] and incorrect Type I error control [3]. In contrast, the empirical standard deviations of ${\tilde{b}}_{\text{TSLS}}$ and ${\hat{b}}_{\text{IVW}}$ are remarkably stable across the different values of b, remaining close to 0.0113 throughout.

### 3.2 Two‐sample Setting

In this setting, two independent samples are simulated from model (12). For each SNP j=1,…,p, ${\hat{\Gamma }}_{j}$ and its standard error are estimated from sample 1 and ${\hat{\gamma }}_{j}$ and its standard error are estimated from sample 2. Based on these GWAS summary statistics, ${\hat{b}}_{\text{IVW}}$, ${\hat{b}}_{\text{TSTSLS}}$, and ${\hat{b}}_{\text{TSIV}}$ are calculated. Both samples have size 10,000, and 1000 simulation replicates are generated. For each replicate, the point estimate and its standard error are calculated for each method. The results are reported in Table 2.

**TABLE 2:** Simulation results under two‐sample setting for ${\hat{b}}_{\text{IVW}}$, ${\hat{b}}_{\text{TSTSLS}}$, and ${\hat{b}}_{\text{TSIV}}$ with sample size n=10,000 over 1,000 replicates.

b					Coverage of 95% CI	
	Estimator	Estimate	SE^a^	SD^b^	Estimate $\pm z_{0.975}\cdot$ SE	${\tilde{b}}_{\text{TSTSLS}}\pm z_{0.975}\cdot$ SE
0.0	${\hat{b}}_{\text{IVW}}$, naïve SE	−0.0002	0.0111	0.0111	0.9520	0.9520
	${\hat{b}}_{\text{IVW}}$, Consistent SE	−0.0002	0.0111	0.0111	0.9520	0.9520
	${\hat{b}}_{\text{TSTSLS}}$	−0.0002	0.0111	0.0111	0.9520	0.9520
	${\hat{b}}_{\text{TSIV}}$	−0.0002	0.0111	0.0111	0.9520	0.9520
0.2	${\hat{b}}_{\text{IVW}}$, naïve SE	0.1991	0.0136	0.0144	0.9290	0.9400
	${\hat{b}}_{\text{IVW}}$, Consistent SE	0.1991	0.0139	0.0144	0.9330	0.9450
	${\hat{b}}_{\text{TSTSLS}}$	0.1991	0.0139	0.0144	0.9330	0.9450
	${\hat{b}}_{\text{TSIV}}$	0.1991	0.0139	0.0144	0.9330	0.9450
0.4	${\hat{b}}_{\text{IVW}}$, naïve SE	0.3984	0.0166	0.0190	0.9070	0.9380
	${\hat{b}}_{\text{IVW}}$, Consistent SE	0.3984	0.0173	0.0190	0.9250	0.9470
	${\hat{b}}_{\text{TSTSLS}}$	0.3984	0.0173	0.0190	0.9250	0.9470
	${\hat{b}}_{\text{TSIV}}$	0.3984	0.0173	0.0190	0.9250	0.9470
0.8	${\hat{b}}_{\text{IVW}}$, naïve SE	0.7971	0.0230	0.0295	0.8750	0.9230
	${\hat{b}}_{\text{IVW}}$, Consistent SE	0.7971	0.0252	0.0295	0.9100	0.9460
	${\hat{b}}_{\text{TSTSLS}}$	0.7970	0.0252	0.0295	0.9090	0.9460
	${\hat{b}}_{\text{TSIV}}$	0.7970	0.0252	0.0295	0.9090	0.9460

^a^ The SE of ${\hat{b}}_{\text{IVW}}$ is the naïve SE computed using Equation (10).

^b^ The standard deviation across 1,000 replicates.

As expected, all estimates are close to the corresponding generating true value. The results also confirm that ${\hat{b}}_{\text{IVW}}$ is not equivalent to ${\hat{b}}_{\text{TSTSLS}}$, a discrepancy that becomes increasingly apparent as the true value of b increases, for example when b=0.8. Moreover, the naïve standard error of ${\hat{b}}_{\text{IVW}}$ differs substantially from the consistent standard error, the latter being nearly identical to those of ${\hat{b}}_{\text{TSTSLS}}$ and ${\hat{b}}_{\text{TSIV}}$. Overall, the results in Table 2 provide empirical support for the theoretical argument that ${\hat{b}}_{\text{IVW}}$ and ${\hat{b}}_{\text{TSTSLS}}$ are not asymptotically equivalent.

The simulation results also reveal an interesting phenomenon. The coverage of the conventional Wald confidence interval based on GWAS summary statistics tends to be anti‐conservative even when the consistent standard error is used, as shown in the column labeled “Estimate $\pm z_{0.975}\cdot$ SE”. When the same confidence interval is centered at the more efficient estimator ${\tilde{b}}_{\text{TSTSLS}}$, as in the column labeled “${\tilde{b}}_{\text{TSTSLS}}\pm z_{0.975}\cdot$ SE”, the coverage probability improves substantially. Here, ${\tilde{b}}_{\text{TSTSLS}}$ serves as a benchmark because it is obtained from individual‐level data and reduces to ${\hat{b}}_{\text{TSTSLS}}$ under Assumption 4. The underlying cause of this phenomenon warrants further investigation.

Recall that the naïve variance of ${\hat{b}}_{\text{IVW}}$ is not consistent. The consistent variance estimator is

$$\hat{\mathrm{Var}}\;({\hat{b}}_{\text{IVW}})=\underset{j}{\sum }\frac{w_{j}^{2}}{{\hat{\gamma }}_{j}^{2}}\left[\hat{\mathrm{Var}}\;({\tilde{\Gamma }}_{j})+{\hat{b}}_{\text{IVW}}^{2}\hat{\mathrm{Var}}\;({\tilde{\gamma }}_{j})\right],\;\;\;w_{j}=\frac{{\hat{\gamma }}_{j}^{2}}{\hat{\mathrm{Var}}\;({\hat{\Gamma }}_{j})}.$$

To better understand the difference between the naïve and consistent variance estimators, we evaluate both quantities directly under the simulation model in Equation (12) using the true parameter values:

(13) $$\mathrm{Var}({\hat{b}}_{\text{IVW}})=\underset{j}{\sum }\frac{w_{j}^{2}}{\gamma_{j}^{2}}\left[\mathrm{Var}({\tilde{\Gamma }}_{j})+b^{2}\mathrm{Var}({\tilde{\gamma }}_{j})\right],\;\;\;w_{j}=\frac{\gamma_{j}^{2}}{\mathrm{Var}({\hat{\Gamma }}_{j})}.$$

The quantities $\mathrm{Var}({\hat{\Gamma }}_{j})$, $\mathrm{Var}({\tilde{\Gamma }}_{j})$, and $\mathrm{Var}({\hat{\gamma }}_{j})$ are also evaluated directly from the simulation model. Similarly, the naïve variance estimator is

$$Var_{\text{naïve}}({\hat{b}}_{\text{IVW}})=\underset{j}{\sum }\frac{w_{j}^{2}}{\gamma_{j}^{2}}\mathrm{Var}({\hat{\Gamma }}_{j}).$$

Figure 3 plots $\mathrm{Var}({\hat{b}}_{\text{IVW}})$ and $Var_{\text{naïve}}({\hat{b}}_{\text{IVW}})$ as functions of b. Consistent with the pattern observed in Table 2, the naïve variance estimator is smaller than the consistent variance estimator throughout the range b∈(0,1). To understand the source of this discrepancy, we also plot the first component of Equation (13)

$$\underset{j}{\sum }\frac{w_{j}^{2}}{\gamma_{j}^{2}}\mathrm{Var}({\tilde{\Gamma }}_{j}),$$

which we refer to as “Component 1”. As discussed earlier, “Component 1” should be smaller than $Var_{\text{naïve}}({\hat{b}}_{\text{IVW}})$, and this relationship is clearly visible in Figure 3. Therefore, the difference $\mathrm{Var}({\hat{b}}_{\text{IVW}})-Var_{\text{naïve}}({\hat{b}}_{\text{IVW}})$ is entirely attributable to the second term in Equation (13)

$$b^{2}\underset{j}{\sum }\frac{w_{j}^{2}}{\gamma_{j}^{2}}\mathrm{Var}({\tilde{\gamma }}_{j})=\mathrm{Var}({\hat{b}}_{\text{IVW}})-\underset{j}{\sum }\frac{w_{j}^{2}}{\gamma_{j}^{2}}\mathrm{Var}({\tilde{\Gamma }}_{j}).$$

This decomposition illustrates that the underestimation of variance by the naïve IVW formula arises from ignoring the uncertainty in the estimated SNP‐exposure effects ${\tilde{\gamma }}_{j}$. The impact of this component grows with |b|, explaining why the discrepancy between the two variance estimators becomes increasingly pronounced as the causal effect increases.

**FIGURE 3:**
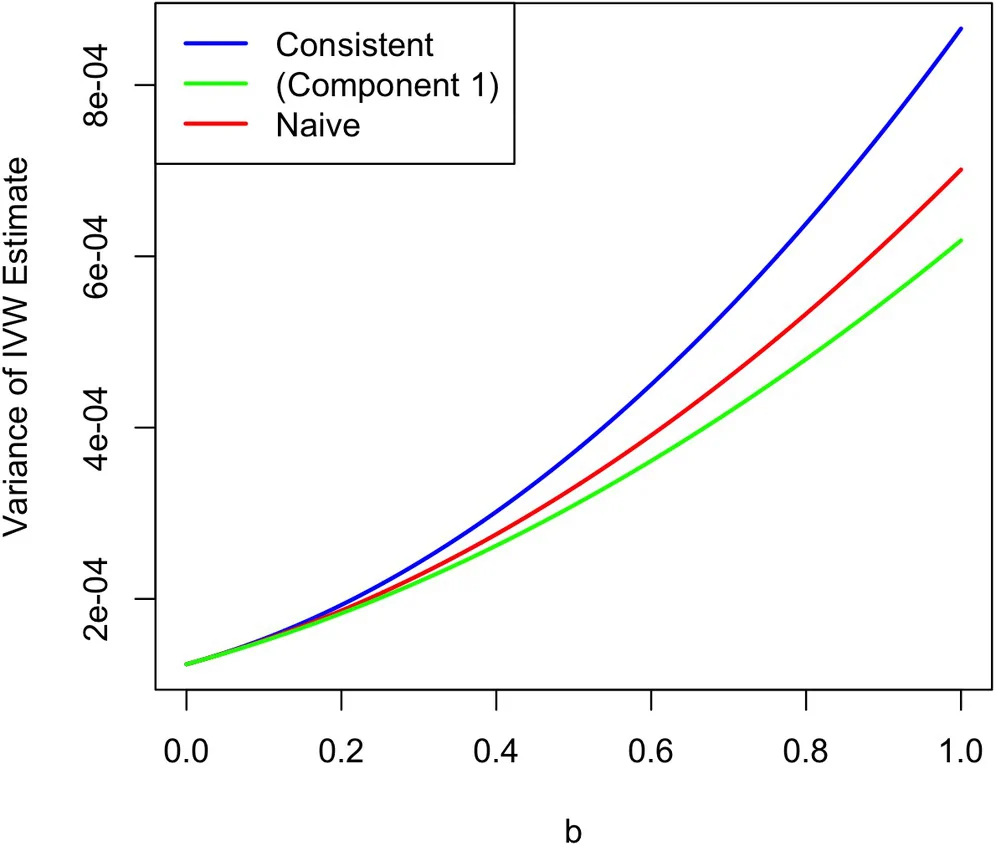
Comparison of the naïve variance estimator and the consistent variance estimator for the simulation model (12) in two‐sample setting. See the text for the meaning of “Component 1”.

## 4 Empirical Example

We illustrate the proposed methods using the bmi.sbp dataset from the R package mr.raps, which was also analyzed by Zhao et al. [14]. The objective is to estimate the causal effect of body mass index (BMI) on systolic blood pressure (SBP). According to the package documentation, the dataset contains GWAS summary statistics from the following three studies:

BMI‐FEM: BMI in females by the Genetic Investigation of ANthropometric Traits (GIANT) consortium (sample size: 171977).
BMI‐MAL: BMI in males in the same study by the GIANT consortium (sample size: 152893).
SBP‐UKBB: SBP using the United Kingdom BioBank (UKBB) data (sample size: 317754).

Thus, two GWASs are available for BMI. To avoid “winner's curse”, BMI‐FEM is used for instrumental selection. Using a significance threshold of $5\times 10^{-8}$ yields 25 instrumental SNPs, whereas a threshold of $1\times 10^{-4}$ yields 160 instrumental SNPs. The corresponding average F‐statistics, which measure instrumental strength, are 33.14 and 9.12, respectively.

In the MR analysis, BMI‐MAL serves as the exposure GWAS, while SBP‐UKBB is used as the outcome GWAS. Table 3 presents the estimates and SEs obtained using three methods: IVW, TSTSLS, and TSIV. Across varying numbers of instrumental SNPs, the estimates from IVW and TSTSLS are consistently similar, with both showing slight deviations from that produced by TSIV. In contrast, the SEs from TSTSLS and TSIV are closely aligned, differing modestly from those of the IVW method.

**TABLE 3:** Analysis of the bmi.sbp data from R package mr.raps.

	25 IV SNPs			160 IV SNPs		
		SE			SE	
Estimator	Estimate	Consistent	Naïve	Estimate	Consistent	Naïve
${\hat{b}}_{\text{IVW}}$	0.33163	0.07484	0.07396	0.31728	0.05448	0.05389
${\hat{b}}_{\text{TSTSLS}}$	0.33162	0.07484		0.31728	0.05448	
${\hat{b}}_{\text{TSIV}}$	0.33155	0.07484		0.31674	0.05448	

This pattern is consistent with the theoretical results developed in the previous sections. In particular, the SNP heritabilities are extremely small for both BMI and SBP. For the analysis based on 160 instrumental SNPs, the SNP heritabilities range from $9.93\times 10^{-9}$ to $1.41\times 10^{-3}$ for BMI, and from $1.15\times 10^{-9}$ to $3.81\times 10^{-4}$ for SBP. The sum of the SNP heritabilities is $9.55\times 10^{-3}$ for BMI and is $2.22\times 10^{-3}$ for SBP. Consequently, the correlations among the marginal GWAS effect estimates are almost negligible (see Equation (8)), which explains why the naïve and consistent variance estimators for IVW are similar in this application.

## 5 Final Remarks

This study tackles the challenge of drawing valid inferences in summary statistics MR without relying on assumptions like the NOME condition and the independence of summary statistics across instrumental SNPs that are known to be violated. Although these issues may be subtle, they have significant implications and have been largely overlooked or inadequately addressed in the existing literature. These issues are closely interrelated. To disentangle them, we examined several widely used MR methods, beginning with the well‐established TSLS method. We derived expressions for TSLS and TSTSLS, including their standard errors, in terms of GWAS summary statistics.

We identify two fallacies in the standard calculation of the IVW standard error. The first concerns the variance of the Wald ratio estimator, ${\hat{\Gamma }}_{j}/{\hat{\gamma }}_{j}$, where the NOME assumption neglects the variability in ${\hat{\gamma }}_{j}$. The second relates to the variance of the IVW estimator, ${\hat{b}}_{\text{IVW}}$, which should be computed conditional on all instrumental SNPs jointly. In contrast, the naïve variance, calculated as $\sum_{j}w_{j}^{2}/{\hat{\gamma }}_{j}^{2}\mathrm{Var}({\hat{\Gamma }}_{j})$, assumes $Cov({\hat{\Gamma }}_{j},{\hat{\Gamma }}_{k})=0$ for j≠k and calculates $\mathrm{Var}({\hat{\Gamma }}_{j})$ conditional only on SNP j. This assumption is not only inconsistent with the underlying model but also unnecessary. When conditioning on the full set of instrumental SNPs, one naturally obtains $Cov({\tilde{\Gamma }}_{j},{\tilde{\Gamma }}_{k})=0$ for j≠k. Failure to make this distinction leads to an inconsistent variance estimator.

Another way to understand why $Cov({\hat{\Gamma }}_{j},{\hat{\Gamma }}_{k})=0$ for j≠k is inconsistent with the underlying model is to notice that, in those marginal regressions, the exposure is the same even though the SNPs are in linkage equilibrium. This term may be small but does not vanish, even asymptotically.

Our simulation results highlight a limitation of using summary statistics in MR: the resulting confidence intervals tend to be anti‐conservative. This anti‐conservatism arises from the common practice of ignoring the off‐diagonal elements of $Z^{\prime }Z$, as specified in Assumption 4. This assumption influences both the point estimate and its SE. Our finding suggests that the primary driver of the reduced coverage probability is the estimate, not the SE.

Our results are based on summary statistics from GWASs that use linear models. For GWASs based on other models, such as logistic regression, further investigation is warranted.

As in many other methods, the common assumption that the instrumental SNPs are in linkage equilibrium is made. One reviewer pointed out that there exists a version of IVW that does not require this assumption [27].

The two‐sample summary statistics TSTSLS, TSIV, and IVW (with consistent SE) have been implemented in an R package iGasso via function tssmr.

## Author Contributions

K.W. conceived and conducted the experiments. K.W. wrote and reviewed the manuscript. G.Z.W. analyzed data for the empirical example and reviewed the manuscript.

## Funding

This work was supported by Environmental Health Sciences Research Center, P30 ES005605.

## Conflicts of Interest

The authors declare no conflicts of interest.

## Data Availability

The data that support the findings of this study are available in GitHub at https://github.com/qingyuanzhao/mr.raps. These data were derived from the following resources available in the public domain:‐mr.raps R package, https://github.com/qingyuanzhao/mr.raps.
